# Correction: Variability in patient sociodemographics, clinical characteristics, and healthcare service utilization among 107,302 treatment seeking smokers in Ontario: A cross-sectional comparison

**DOI:** 10.1371/journal.pone.0241894

**Published:** 2020-11-03

**Authors:** Dolly Baliunas, Laurie Zawertailo, Sabrina Voci, Evgenia Gatov, Susan J. Bondy, Longdi Fu, Peter L. Selby

The ORCID iDs are missing for the second, fifth, and seventh author. Please see the authors’ respective ORCID iDs here:

- Author Laurie Zawertailo’s ORCID iD is: 0000-0002-4547-1565 (https://orcid.org/0000-0002-4547-1565).
- Author Susan J. Bondy’s ORCID iD is: 0000-0002-6516-4159 (https://orcid.org/0000-0002-6516-4159).
- Author Peter L. Selby’s ORCID iD is 0000-0001-5401-2996 (https://orcid.org/0000-0001-5401-2996).

## References

[1] Baliunas D, Zawertailo L, Voci S, Gatov E, Bondy SJ, Fu L, et al (2020) Variability in patient sociodemographics, clinical characteristics, and healthcare service utilization among 107,302 treatment seeking smokers in Ontario: A cross-sectional comparison. PLoS ONE 15(7): e0235709 10.1371/journal.pone.0241894 32650339 PMC7351500

