# Supplementary material for: Variability in patient sociodemographics, clinical characteristics, and healthcare service utilization among 107,302 treatment seeking smokers in Ontario: A cross-sectional comparison
Source: PLoS One. 2020 Jul 10;15(7):e0235709. doi: 10.1371/journal.pone.0235709 (PMC7351500; doi:10.1371/journal.pone.0235709)
Supplement: S1 Appendix — (PDF) [file pone.0235709.s001.pdf]

S1 Appendix: Prevalent comorbidity algorithms and associated validation papers

Comorbidity	Algorithm	Validation	Citation
Asthma	≥1 hospitalization *or* ≥2 physician billings in a two-year period	80.6% sensitivity 81.4% specificity	Gershon AS, Wang C, Guan J, Vasilevska-Ristovska J, Cicutto L, To T. Identifying patients with physician-diagnosed asthma in health administrative databases. *Canadian respiratory journal.* 2009;16(6):183-188.
Coronary heart failure (CHF)	≥1 hospitalization *or* 1 physician billing / ED visit, *followed by* ≥1 hospitalization / ED visit / physician billing within one year	84.8% sensitivity 97.0% specificity	Schultz S, Rothwell D, Chen Z, Tu K. Identifying cases of congestive heart failure from administrative data: a validation study using primary care patient records. *Chronic diseases and injuries in Canada.* 2013;33(3).
Chronic obstructive pulmonary disease (COPD)	≥1 hospitalization *or* ≥1 physician billing	85.0% sensitivity 78.4% specificity	Gershon A, Wang C, Guan J, Vasilevska-Ristovska J, Cicutto L, To T. Identifying individuals with physician diagnosed COPD in health administrative databases. *COPD*. 2009;6:388-94.
Diabetes mellitus	≥2 physician billings *or* ≥1 drug claim in a one-year period *or* ≥1 hospitalization	90.0% sensitivity 97.7% specificity	Hux JE, Ivis F, Flintoft V, Bica A. Diabetes in Ontario: determination of prevalence and incidence using a validated administrative data algorithm. *Diabetes care.* 2002;25(3):512-516.
Hypertension	≥1 hospitalization *or* ≥2 physician billings in a two-year period.	72% sensitivity 95% specificity	Tu K, Campbell NR, Chen Z-L, Cauch-Dudek KJ, McAlister FA. Accuracy of administrative databases in identifying patients with hypertension. *Open medicine.* 2007;1(1):e18.
Myocardial infarction	≥1 hospitalization	88.8% sensitivity 92.8% specificity	Austin PC, Daly PA, Tu JV. A multicenter study of the coding accuracy of hospital discharge administrative data for patients admitted to cardiac care units in Ontario. *American heart journal.* 2002;144(2):290-296.

Abbreviations: ED, emergency department.
