# Supplementary material for: Variability in patient sociodemographics, clinical characteristics, and healthcare service utilization among 107,302 treatment seeking smokers in Ontario: A cross-sectional comparison
Source: PLoS One. 2020 Jul 10;15(7):e0235709. doi: 10.1371/journal.pone.0235709 (PMC7351500; doi:10.1371/journal.pone.0235709)
Supplement: S2 Appendix — (PDF) [file pone.0235709.s002.pdf]

S2 Appendix: Population-based health administrative datasets used for cost ascertainment

Utilization Database[a]	Description	Types of services
Patient Agency Program Enrolment (CAPE)	Registry of patients enrolled in a primary care model. Includes information on program type (family health team, family health organization, family health network, etc.) and patient enrolment status.	Capitation costs
Continuing Care Reporting System (CCRS)	Contains information about residents receiving facility-based continuing care services. Range of services includes complex continuing care, extended or chronic care, and residential care providing nursing services (i.e., long-term care).	Complex continuing care admissions, long-term care
Discharge Abstract Database (CIHI-DAD)	Contains demographic, clinical, and administrative data for inpatient hospital admissions (patient separations).	Inpatient hospitalizations
Home Care Database (HCD), Ontario Home Care Administrative System (OHCAS)	Captures information on all services provided or coordinated by Ontario Community Care Access Centres, including patient data, intake and assessment information, admission and discharge, etc.	Home care services
National Ambulatory Care Reporting System (NACRS)	Contains data from hospital- and community-based ambulatory care services, including day surgery, outpatient clinics, and emergency departments.	Emergency department visits, dialysis and oncology clinic visits
National Rehabilitation Reporting System (NRS)	Contains patient data from adult inpatient rehabilitation facilities, such as administrative data (referral, admission, and discharge) and health/functional characteristics.	Rehabilitation admissions
Ontario Health Insurance Plan (OHIP)	Contains claims paid by OHIP for services provided by all eligible healthcare providers, including physicians (primary and specialist physicians), groups, and laboratories.	Outpatient physician visits, laboratory, and non-physician services
Ontario Drug Benefit (ODB)	Contains claims for prescription drugs covered under the ODB program. Primarily includes drug claims for individuals 65 years of age and older, but also coverage under special ODB programs (e.g., social assistance).	Medication use
Ontario Mental Health Reporting System (OMHRS)	Contains data on patients in adult designated inpatient mental health beds in acute and psychiatric facilities (admission and discharge dates, diagnoses, service utilization, etc).	Mental health admissions

[a]Datasets were linked using unique encoded identifiers and analyzed at ICES.

Sources: Wodchis WP, Bushmeneva K, Nikitovic M, McKillop I. Guidelines on Person Level Costing Using Administrative Databases in Ontario. Working Paper Series. Vol 1. Toronto: Health System Performance Research Network; 2013. Available from: https://hspn.ca/wp-content/uploads/2019/09/Guidelines_on_PersonLevel_Costing_May_2013.pdf; Desveaux L, Agarwal P, Shaw J, Hensel JM, Mukerji G, Onabajo N, et al. A randomized wait-list control trial to evaluate the impact of a mobile application to improve self-management of individuals with type 2 diabetes: a study protocol. BMC Med Inform Decis Mak. 2016;16(1):144.
