# Supplementary material for: Variability in patient sociodemographics, clinical characteristics, and healthcare service utilization among 107,302 treatment seeking smokers in Ontario: A cross-sectional comparison
Source: PLoS One. 2020 Jul 10;15(7):e0235709. doi: 10.1371/journal.pone.0235709 (PMC7351500; doi:10.1371/journal.pone.0235709)
Supplement: S3 Appendix — (PDF) [file pone.0235709.s003.pdf]

S3 Appendix: Mental health and addictions-related outcomes

Mental health and addictions-related outpatient physician visit definition (OHIP): DSM-IV	
Psychiatrist visit	Any outpatient (office, home, long term care) non-lab visit/consult to a psychiatrist.
Mental health and addictions-related general practitioner/family physician visit	Any outpatient (office, home, long term care) non-lab visit/consult to a general practitioner/family physician and a mental health diagnostic code (295, 296, 297, 298, 300, 301, 302, 306, 309, 311, 303, 304, 897, 898, 899, 900, 901, 902, 904, 905, 906, 909).
**Mental health and addictions-related emergency department visits (NACRS) and hospitalizations (CIHI-DAD): ICD-10-CA codes[b]**	
Any mental health and addictions	F04-F99 in primary problem field or X60-X84, Y10-Y19, Y28 in secondary positions when there is no F04-F99 code in the primary problem field.
**Hospitalizations in designated psychiatric beds (OMHRS): DSM-IV codes[a]**	
Any mental health and addictions	Any (including missing diagnoses). Exclude 290.x. or 294.x (Dementia) in primary problem field or provisional diagnoses 2 when primary problem field is blank.

Abbreviations: ICD-10-CA, International Classification of Diseases, 10th edition with Canadian enhancements; DSM-IV, Diagnostic and Statistical Manual, 4th revision. Datasets: Ontario Health Insurance Plan (OHIP); National Ambulatory Care Reporting System (NACRS); Canadian Institute for Health Information’s Discharge Abstract Database (CIHI-DAD); Ontario Mental Health Reporting System (OMHRS).

[a]Diagnostic codes for dementia were excluded, as they represent a unique patient population that was not the focus of this study.
