# Supplementary material for: Variability in patient sociodemographics, clinical characteristics, and healthcare service utilization among 107,302 treatment seeking smokers in Ontario: A cross-sectional comparison
Source: PLoS One. 2020 Jul 10;15(7):e0235709. doi: 10.1371/journal.pone.0235709 (PMC7351500; doi:10.1371/journal.pone.0235709)
Supplement: Attachment — Submitted filename: Response to Reviewers.docx [file pone.0235709.s004.docx]

**Response to Reviewers**

Journal requirements:

When submitting your revision, we need you to address these additional requirements:

1.    Please ensure that your manuscript meets PLOS ONE's style requirements, including those for file naming. The PLOS ONE style templates can be found at <http://www.plosone.org/attachments/PLOSOne_formatting_sample_main_body.pdf> and <http://www.plosone.org/attachments/PLOSOne_formatting_sample_title_authors_affiliations.pdf>

**Response:** We have reviewed the revised manuscript and made appropriate edits to ensure that it meets PLOS ONE’s style requirements.

2. Please ensure you have included the registration number for the clinical trial referenced in the manuscript.

**Response:** As the paper does not report on a clinical trial, we have assumed that the change requested here is in reference to the two clinical trial protocols we cited (lines 393-396; line numbers refer to revised manuscript with tracked changes) and have added the registration numbers for both trials in the text. In addition, the results of one of these trials was recently published, so we have updated that citation to refer readers to the paper reporting on trial outcomes. Finally, the word “currently” was removed from this sentence, given that both of these trials are now closed.

3. Thank you for stating the following in the Competing Interests section:

"I have read the journal's policy and the authors of this manuscript have the following competing interests: DB reports grants from the Canadian Cancer Society Research Institute during the conduct of the study; she also reports grants from a Pfizer GRAND award, outside the submitted work. LZ reports grants from the Canadian Cancer Society Research Institute during the conduct of the study; she also reports grants from Pfizer GRAND Awards, outside the submitted work. PLS reports receiving grants from the Centre for Addiction and Mental Health, Ontario Ministry of Health and Long-Term Care, Health Canada, Canadian Institutes of Health Research, Canadian Cancer Society Research Institute, Canadian Partnership Against Cancer, Medical Psychiatry Alliance, Ontario Neurotrauma Foundation, the Patient-Centered Outcomes Research Institute, Bhasin Consulting Fund Inc and Pfizer Inc./Canada. PLS also reports serving on advisory boards and receiving consulting fees and honoraria from Pfizer Canada Inc., Johnson & Johnson Group of Companies, Evidera Inc., Miller Medical Communications, NVision Insight Group, Myelin & Associates, University of Ottawa Heart Institute, Royal College of Physicians and Surgeons of Canada, Royal Victoria Regional Health Centre, the University of Toronto Department of Family and Community Medicine, Northern Ontario School of Medicine, Canadian Partnership Against Cancer, Battle River Treaty 6 Healthcare, Lung Association of Nova Scotia, Exchange Summit, Toronto Public Health, Ontario Association of Public Health Dentistry and ECHO. PLS also reports that MedPlan Communications assisted in organizing Pfizer Canada Inc. Advisory Board events for which he was a consultant and that Pfizer Inc., Novartis, and Johnson & Johnson are vendors of record for providing free/discounted smoking cessation pharmacotherapy for research studies on which he is principal or co-investigator. No other author has competing interests to declare."

Please confirm that this does not alter your adherence to all PLOS ONE policies on sharing data and materials, by including the following statement: "This does not alter our adherence to  PLOS ONE policies on sharing data and materials.” (as detailed online in our guide for authors [http://journals.plos.org/plosone/s/competing-interests](about:blank)).  If there are restrictions on sharing of data and/or materials, please state these. Please note that we cannot proceed with consideration of your article until this information has been declared.

Please include your updated Competing Interests statement in your cover letter; we will change the online submission form on your behalf.

Please know it is PLOS ONE policy for corresponding authors to declare, on behalf of all authors, all potential competing interests for the purposes of transparency. PLOS defines a competing interest as anything that interferes with, or could reasonably be perceived as interfering with, the full and objective presentation, peer review, editorial decision-making, or publication of research or non-research articles submitted to one of the journals. Competing interests can be financial or non-financial, professional, or personal. Competing interests can arise in relationship to an organization or another person. Please follow this link to our website for more details on competing interests: [http://journals.plos.org/plosone/s/competing-interests](about:blank)

**Response:** We have updated our Competing Interests statement and included it in our cover letter.

4. We note that you have indicated that data from this study are available upon request. PLOS only allows data to be available upon request if there are legal or ethical restrictions on sharing data publicly. For information on unacceptable data access restrictions, please see <http://journals.plos.org/plosone/s/data-availability#loc-unacceptable-data-access-restrictions>.

In your revised cover letter, please address the following prompts:

a) If there are ethical or legal restrictions on sharing a de-identified data set, please explain them in detail (e.g., data contain potentially identifying or sensitive patient information) and who has imposed them (e.g., an ethics committee). Please also provide contact information for a data access committee, ethics committee, or other institutional body to which data requests may be sent.

b) If there are no restrictions, please upload the minimal anonymized data set necessary to replicate your study findings as either Supporting Information files or to a stable, public repository and provide us with the relevant URLs, DOIs, or accession numbers. Please see <http://www.bmj.com/content/340/bmj.c181.long> for guidelines on how to de-identify and prepare clinical data for publication. For a list of acceptable repositories, please see <http://journals.plos.org/plosone/s/data-availability#loc-recommended-repositories>.

We will update your Data Availability statement on your behalf to reflect the information you provide.

**Response**: We have provided a response to this item in the cover letter as requested.

Reviewers' comments:

Reviewer's Responses to Questions

**Comments to the Author**

1. Is the manuscript technically sound, and do the data support the conclusions?

The manuscript must describe a technically sound piece of scientific research with data that supports the conclusions. Experiments must have been conducted rigorously, with appropriate controls, replication, and sample sizes. The conclusions must be drawn appropriately based on the data presented.

Reviewer #1: Yes

Reviewer #2: Yes

2. Has the statistical analysis been performed appropriately and rigorously?

Reviewer #1: Yes

Reviewer #2: Yes

3. Have the authors made all data underlying the findings in their manuscript fully available?

The [PLOS Data policy](http://www.plosone.org/static/policies.action#sharing) requires authors to make all data underlying the findings described in their manuscript fully available without restriction, with rare exception (please refer to the Data Availability Statement in the manuscript PDF file). The data should be provided as part of the manuscript or its supporting information, or deposited to a public repository. For example, in addition to summary statistics, the data points behind means, medians and variance measures should be available. If there are restrictions on publicly sharing data—e.g. participant privacy or use of data from a third party—those must be specified.

Reviewer #1: No

Reviewer #2: Yes

4. Is the manuscript presented in an intelligible fashion and written in standard English?

PLOS ONE does not copyedit accepted manuscripts, so the language in submitted articles must be clear, correct, and unambiguous. Any typographical or grammatical errors should be corrected at revision, so please note any specific errors here.

Reviewer #1: Yes

Reviewer #2: Yes

**Response:** We would like to note that we have reviewed the paper fully and made minor edits throughout the manuscript that are tracked, including (but not limited to) the following:

- We have updated the corresponding author’s e-mail address (line 21)
- We edited “Off label use permitted” to “Dosing as per clinical discretion” in Table 1 for improved clarity (NRT column for FHT, NPLC, CHC-B, and AA models)
- We have added an “Ethics” heading on line 197 to more clearly separate the information on ethics approval and consent from information on the analysis
- We deleted information in a footnote for Figure 1 that was not needed and may not be clear for readers outside Ontario (lines 242-243).

5. Review Comments to the Author

Please use the space provided to explain your answers to the questions above. You may also include additional comments for the author, including concerns about dual publication, research ethics, or publication ethics. (Please upload your review as an attachment if it exceeds 20,000 characters)

Reviewer #1: This is a well-written paper about the STOP smoking cessation program in Ontario, Canada and its 11 specific sub-programs. The manuscript describes the variability in patient sociodemographic and clinical characteristics, and in healthcare service utilization.

Comments are in order of appearance.

Around line 15 in the introduction, please consider adding that it has been shown that NRT and counselling offered together are more effective than NRT alone.

**Response:** We thank the reviewer for this suggestion. We have added a sentence where suggested (now lines 70-71), with appropriate citation.

When mentioning all the different data sources, starting in line 67, please include references to all of them, not just the ones you list in Appendix 2. If you do not want to include the information in the references, then just add them to Appendix 2. Some readers might want to follow up.

**Response:** We have now provided references for further information about all of the datasets in the Data Sources section.

Footnotes to Table 2: “** suppressed due to small cell count (<6)” should not be a category between * and *** which are about the proportions of missing values. How about giving the small cell count an entirely different symbol?

**Response:** We have taken the reviewer’s suggestion and now use an entirely different symbol (†) to denote that data was suppressed due to small cell count; the symbol denoting proportion of missing > 10% is now **.

Please spell out MHA the first time it appears in the text, it is only spelled out in one of the table legends.

**Response:** Since MHA was only mentioned twice in the text, we have removed the abbreviation and spell it out fully.

The manuscript has a lot of detail about the different Ontario programs and about the characteristics of those enrolled in them. This information is valuable for future smoking cessation programs trying to reach different populations of smokers in this very specific region. What would add to the value of the paper would be somewhat more of a discussion of how this might (or might not) be generalizable outside of Ontario. Can you make any recommendations for readers who are not in Ontario or not even in Canada?

**Response:** We have added a paragraph to the Discussion (lines 397-405) to review the issue of generalizability of the findings outside of Ontario, particularly in light of the fact that Ontario has a universal health care system. We recommend that implementers consider how contextual factors might impact healthcare access disparities in their own local setting/s.

Figures: Figures should be more self-explanatory. Please consider the full names of the 11 sub-programs in the legend, including the years in which they were active.

**Response:** We have taken the reviewer’s suggestion to make the figures more clear by adding the full names of all 11 treatment models in the legend, along with the years in which they operated.

Reviewer #2: This is an interesting and well-written manuscript describing sociodemographic, clinical and healthcare services utilization among smokers seeking smoking cessation services in Ontario Canada. The authors found that there were differences in the case-mix of populations that were reached by different cessation models, but less clear were the implication of these findings and how they could promote equity in access to cessation programs. I highlight some opportunities for clarifications below.

Abstract:
- The abstract would be clearer if the authors described in the background the different models of cessation care that are available to patients through the STOP program. While a long description is not necessary, a short description describing general approaches such as in-person, telephone etc. would be helpful.

**Response:** We revised the Background section of the Abstract to provide some indication of the general approaches to treatment, and their variation, across the 11 different treatment models.

- It is also not clear how the authors came up with the 107,302 case mix. For e.g., is this from a primary care populations and what clinics/hospitals were represented in this population. A brief description of the fact that the 107,302 people represent people who engaged in the STOP program would be helpful.

**Response:** We have revised the relevant sentences in the Abstract to make it clear that the sample of 107,302 were patients that enrolled in STOP program between 18Oct2005 and 31Mar2016, across all 11 different models.

- The implications could be made clearer in terms of models that may be most effective for subpopulations with specific comorbidities, versus a more general population-based strategy to address tobacco use

**Response:** We have revised the Discussion section of the Abstract to clarify the type of models that may be more effective for subpopulations with comorbidities.

Introduction
- The introduction is really clear in terms of its objective.

- My only suggestion is to provide a brief description of the 11 models or at least refer the reader to the Table and say that some were initiated by a referral (??) and some were self-referred. And who they were intended to target at the time of inception. i.e., were some models specifically more intensive because they were seeing a higher risk population (e.g., people in addition treatment). While some of this is described in the methods, I might bring some of this info up into the intro or at least refer the reader to the Table 1. I think some clarity on these models in the text would really help because when you get the results, it gets very confusing to keep all the models straight.

**Response:** We can appreciate that it is hard to keep track of all of the models and thank the reviewer for suggestions on how to make this clearer for the reader. We now refer the reader to Table 1 in the Introduction on line 79. We also have noted that patients could either self-refer or be referred from a healthcare practitioner (line 81). Additionally, we have added a few sentences to provide more background on the types of models offered; including mention that clinic-based treatment did evolve over time to provide more personalized treatment, allowing for more intensive support when needed (lines 81-90). We wish to clarify, however, that the ability to offer patients with more complex needs personalized or more intensive treatment did not differ between AA, FHT, CHC and NPLC models.

Methods
- Why was a long-term costs approach not considered in this study, i.e., costs past the index visit?

**Response:** We thank the reviewer for raising this question. Indeed, an examination of healthcare costs incurred subsequent to treatment in the STOP program are of great interest to our research team, and is the focus of separate research studies that are currently being undertaken. However, the specific objective of the current study was to describe and compare patients at the time at which they initiated smoking cessation treatment. As such, we decided that the total healthcare costs accrued during the 2-year period immediately prior to enrollment best reflected recent healthcare costs.

- Do the different STOP models for cessation, self-select for specific populations. For e.g., the web-based model may be best directed to a group that may not be very sick, whereas the addiction treatment model may target a higher needs group. If so, then costs would be vastly different based on the population seeking cessation treatment from a specific model. What information is cost adding in this analysis?

**Response:** Given patients were not allocated randomly, and some of the models did target higher needs populations (e.g., addictions agency and tertiary care models), we did expect that patients enrolling in these models would have incurred higher healthcare costs. However, we felt it important to examine this data to confirm our hypothesis, and to assess the relative difference between models.

- How was missing data treated?

**Response:** The proportion of missing data on individual characteristics was calculated for each model separately; less than 5%, missing data on a characteristic was ignored; 5-10% missing data on a characteristic is noted using a symbol in the relevant table; and where greater than 10% of data was missing, the characteristic was not reported for that model. We have added this information to the Methods section (lines 188-191), as previously, it was only conveyed using symbols in the Table 2 footnotes.

Results
- The most difficult part of the results is keeping the models straight and simplifying the results in the text so that it’s clear what the primary characteristics are for each of the models. One suggestion is to not present as many characteristics in the text but only emphasize the key ones so that the reader comes out with some key findings.

**Response:** We can appreciate that the results may be challenging to follow given the number of models. Thus, we have taken the reviewer’s suggestion and made edits throughout the Results section to simplify the text and make key findings more apparent for readers.

- Similarly Table 2 is really long and has a lot of information. Could you divide this into two tables?

**Response:** We have separated this table and now present patient comorbidity data in Table 2 and healthcare utilization data in Table 3.

- Also the sample sizes for some of the models are quite small, example CHC or nurse practitioner led clinics – is there utility in combining some of these smaller models? Or is the reasoning to keep them separate because they were intended to target specific populations.

**Response:** Where models were similar in both service model and intended patient population, we did indeed combine models for this study. For example, both the Web and Telephone models were combinations of smaller models. Other models were not combined (i.e., CHC and NPLC), because it was of interest to compare different service models.

Discussion
- The discussion is well-written. The main questions that I have that can be addressed in the discussion:

o whether these models catered to specific sub-populations at the outset and if that has changed over time.

**Response:** We have made an addition to lines 329-330 to note that treatment was not modified for each model in an effort to target different sub-populations.

o Are there cessation outcomes data available for patients enrolled in each of the STOP models. If there are, do the cessation rates reflect characteristics of the population seeking specific models?

**Response:** We do attempt to contact all individuals who enroll in each of the STOP models to ascertain quit outcome, and as such, have outcome data available for those who responded to a follow-up survey. As the purpose of this initial study was to describe and compare patients at the time of enrollment in the program, we have not linked outcome data with the enrollment and health-administrative data presented in this paper. Examination of cessation outcomes is of great interest and importance, and may be undertaken in a future study. However, given the variation between models in treatment offered, setting, years of operation, and follow-up response rate, we are limited in our ability to infer to what extent any differences in cessation rate reflect the characteristics of the populations reached.

o How much is reach defined by what is currently available and not what can be made available? i.e., if there were more access to web-based/telephone models in rural setting would there be equal reach compared to the NP based models. What can this study inform us about models that might be most impactful for low-income populations in Ontario.

**Response:** We thank you for this thoughtful question. We acknowledge that the case-mix of patients served by the different STOP models has been constrained by what has been available at the time, as opposed to what would be theoretically possible. For example, the Web and Telephone models were theoretically available to all eligible smokers in Ontario and were advertised via population-based media strategies. If however, targeted media strategies were employed to raise awareness in particular sub-groups, the patient case mix may have been different. We have added a sentence to the discussion section to describe this (lines 334-336).

6. PLOS authors have the option to publish the peer review history of their article ([what does this mean?](https://journals.plos.org/plosone/s/editorial-and-peer-review-process#loc-peer-review-history)). If published, this will include your full peer review and any attached files.

If you choose “no”, your identity will remain anonymous but your review may still be made public.

**Do you want your identity to be public for this peer review?** For information about this choice, including consent withdrawal, please see our [Privacy Policy](https://www.plos.org/privacy-policy).

Reviewer #1: No

Reviewer #2: No

[NOTE: If reviewer comments were submitted as an attachment file, they will be attached to this email and accessible via the submission site. Please log into your account, locate the manuscript record, and check for the action link "View Attachments". If this link does not appear, there are no attachment files to be viewed.]

While revising your submission, please upload your figure files to the Preflight Analysis and Conversion Engine (PACE) digital diagnostic tool, <https://pacev2.apexcovantage.com/>. PACE helps ensure that figures meet PLOS requirements. To use PACE, you must first register as a user. Registration is free. Then, login and navigate to the UPLOAD tab, where you will find detailed instructions on how to use the tool. If you encounter any issues or have any questions when using PACE, please email us at [figures@plos.org](mailto:figures@plos.org). Please note that Supporting Information files do not need this step.
