# Supplementary material for: Variability in patient sociodemographics, clinical characteristics, and healthcare service utilization among 107,302 treatment seeking smokers in Ontario: A cross-sectional comparison
Source: PLoS One. 2020 Jul 10;15(7):e0235709. doi: 10.1371/journal.pone.0235709 (PMC7351500; doi:10.1371/journal.pone.0235709)
Supplement: Attachment — Submitted filename: Response to Reviewers.docx [file pone.0235709.s005.docx]

You have done a nice job of responding to the reviewers' comments.

I only have one small request.  In the conclusion of the abstract you have vague statements that the healthcare system should ensure "some variation" in the cessation models used depending on patient and context.  The paper would be a lot better if you can make these statements more specific and concrete.  You have the relevant information in the Discussion.

**Response:** We have revised the Discussion section of the Abstract to address your requested change, and made some further revision to the remainder of the Abstract in order to meet the word count limit.
